# Retraction: A Stress Self‐Adaptive Silicon/Carbon “Ordered Structures” to Suppress the Electro‐Chemo‐Mechanical Failure: Piezo‐Electrochemistry and Piezo‐Ionic Dynamics

**DOI:** 10.1002/advs.202412738

**Published:** 2024-10-28

## Abstract

H. Zhao, K. Liang, S. Wang, Z. Ding, X. Huang, W. Chen, Y. Ren, and J. Li, “A Stress Self‐Adaptive Silicon/Carbon “Ordered Structures” to Suppress the Electro‐Chemo‐Mechanical Failure: Piezo‐Electrochemistry and Piezo‐Ionic Dynamics,” *Advanced Science* 10, no. 29 (2023): 2303696.

https://doi.org/10.1002/advs.202303696

The above article, published online on 21 August 2023 in Wiley Online Library (wileyonlinelibrary.com), has been retracted by agreement between the authors; the journal Editor‐in‐Chief, Kirsten Severing; and Wiley‐VCH GmbH. The retraction has been agreed upon following an investigation into concerns raised by a third party, which revealed inappropriate duplication of image panels (Figures 2j, 3h,i, and 6, and Figures S4, S5, and S13a, Supporting Information) between this article and other articles that were previously published in a different scientific context. Due to the number and the level of inconsistencies identified in the published figures, the editors consider the conclusions of this manuscript substantially compromised. The corresponding authors, Y. Ren and J. Li, asked to retract the article due to misused figures, because of which they cannot guarantee the validity of the data and the conclusions.

H. Zhao, K. Liang, S. Wang, Z. Ding, X. Huang, W. Chen, Y. Ren, and J. Li, “A Stress Self‐Adaptive Silicon/Carbon “Ordered Structures” to Suppress the Electro‐Chemo‐Mechanical Failure: Piezo‐Electrochemistry and Piezo‐Ionic Dynamics,” *Advanced Science* 10, no. 29 (2023): 2303696.

https://doi.org/10.1002/advs.202303696

The above article, published online on 21 August 2023 in Wiley Online Library (wileyonlinelibrary.com), has been retracted by agreement between the authors; the journal Editor‐in‐Chief, Kirsten Severing; and Wiley‐VCH GmbH. The retraction has been agreed upon following an investigation into concerns raised by a third party, which revealed inappropriate duplication of image panels (Figures 2j, 3h,i, and 6, and Figures S4, S5, and S13a, Supporting Information) between this article and other articles that were previously published in a different scientific context. Due to the number and the level of inconsistencies identified in the published figures, the editors consider the conclusions of this manuscript substantially compromised. The corresponding authors, Y. Ren and J. Li, asked to retract the article due to misused figures, because of which they cannot guarantee the validity of the data and the conclusions.

